# Evaluation of 1,094 children diagnosed with scabies in Turkey: a 5-year retrospective observational study

**DOI:** 10.1051/parasite/2026041

**Published:** 2026-07-31

**Authors:** Zeynep Üze Okay, Berker Okay, Emanet Çelebi, Şeyma Açıkgöz, Emin Arvas, Mehmet Şefik Avcı, Eda Yıldız, Nahid Ahmadian, Kamil Şahin, Gülşen Akkoç

**Affiliations:** 1 University of Health Sciences, Haseki Training and Research Hospital, Department of Pediatrics Sultangazi Istanbul Turkey; 2 Istanbul University, Istanbul Faculty of Medicine, Department of Pediatrics Fatih Istanbul Turkey; 3 Marmara University Pendik Training and Research Hospital, Department of Pediatric Infectious Diseases Pendik Istanbul Turkey

**Keywords:** Child, Scabies, Crusted scabies, Nodular scabies, Recurrence, Permethrin

## Abstract

Scabies is a common health concern, particularly among infants and children from socioeconomically disadvantaged households. This single-center retrospective observational cohort study describes scabies phenotypes, associated factors, treatment patterns, complications, and 12-month scabies-related re-attendance among diagnosed children between March 2018 and March 2023. Data from 1,094 children living in Turkey were extracted from medical records. Patients were classified as having classic, crusted, or nodular scabies for descriptive analyses; crusted and nodular scabies were combined as variant scabies only for selected regression models. Logistic regression was used to identify factors associated with variant scabies and hospitalization. Most cases occurred in fall and winter (67.6%). Crusted and nodular scabies were identified in 93 (8.5%) and 18 (1.6%) patients, respectively. Underlying abnormalities were reviewed separately for crusted and nodular scabies. Among hospitalized children, 61.5% had variant scabies, and clinically suspected secondary bacterial infection was documented in 77 of 78 hospitalized patients. Cultures were obtained from all hospitalized children; among culture-positive cases, *Staphylococcus aureus* was the most frequently isolated organism. Among children who re-attended within one year, permethrin was the most frequently documented treatment (92.3% of documented treatments). Underlying disease and younger age were independently associated with variant scabies, whereas underlying disease, variant scabies, and younger age were independently associated with hospitalization. Re-attendance was more frequent in variant scabies (*p* < 0.001). Crusted scabies warrants careful evaluation for underlying abnormalities and secondary infection, while nodular scabies should be interpreted as a distinct hypersensitivity-related phenotype. These findings support proactive household-level management and follow-up.

## Abstract (fr)

La gale est un problème de santé fréquent, en particulier chez les nourrissons et les enfants issus de foyers défavorisés sur le plan socio-économique. Cette étude de cohorte observationnelle rétrospective monocentrique décrit les phénotypes de la gale, les facteurs associés, les modalités de traitement, les complications et les motifs de reconsultation dans les 12 mois suivant le diagnostic chez des enfants pris en charge entre mars 2018 et mars 2023. Les données concernant 1 094 enfants résidant en Turquie ont été extraites des dossiers médicaux. Pour les analyses descriptives, les patients ont été classés selon la forme de la maladie : gale classique, gale profuse (ou hyperkératosique/croûteuse) ou gale nodulaire ; les formes profuse et nodulaire ont été regroupées sous le terme de « variantes de la gale » uniquement pour certains modèles de régression. Une régression logistique a été utilisée pour identifier les facteurs associés aux variantes de la gale et à l'hospitalisation. La majorité des cas sont survenus en automne et en hiver (67,6 %). Des formes profuse et nodulaire ont été identifiées chez respectivement 93 (8,5 %) et 18 (1,6 %) patients. Les affections sous-jacentes ont été examinées séparément pour les formes profuse et nodulaire. Parmi les enfants hospitalisés, 61,5 % présentaient une variante de la gale, et une surinfection bactérienne cliniquement suspectée a été documentée chez 77 des 78 patients hospitalisés. Des prélèvements pour culture ont été réalisés chez tous les enfants hospitalisés ; parmi les cultures positives, *Staphylococcus aureus* était le germe le plus fréquemment isolé. Chez les enfants ayant reconsulté dans l'année, la perméthrine était le traitement le plus souvent documenté (92,3 % des traitements recensés). La présence d'une affection sous-jacente et le jeune âge étaient indépendamment associés aux variantes de la gale, tandis que l'affection sous-jacente, les variantes de la gale et le jeune âge étaient indépendamment associés à l'hospitalisation. Le taux de reconsultation était plus élevé pour les variantes de la gale (*p* < 0,001). La gale profuse nécessite une évaluation approfondie à la recherche d'affections sous-jacentes et de surinfections, tandis que la gale nodulaire doit être considérée comme un phénotype distinct lié à une réaction d'hypersensibilité. Ces résultats plaident en faveur d'une prise en charge et d'un suivi proactifs au niveau du foyer.

## Introduction

Human scabies is a contagious parasitic skin disease caused by infestation with *Sarcoptes scabiei* var. *hominis* [2, 31]. Transmission occurs mainly through prolonged direct skin-to-skin contact and may be amplified under conditions such as close infant–caregiver contact, household crowding, poverty, delayed diagnosis, and limited access to healthcare [6, 14, 24, 31]. Accordingly, scabies is particularly common among infants and young children and in socioeconomically disadvantaged or overcrowded communities [6, 24].

Classic scabies typically presents with intense pruritus, papules, excoriations, and/or burrows in a typical distribution, including the interdigital spaces, wrists, axillae, trunk/waistline, and genital area [7, 17]. Crusted scabies and nodular scabies are less common but clinically distinct manifestations. Crusted scabies is characterized by hyperkeratotic plaques, thick crusting, fissuring, and a markedly increased mite burden, and is most often associated with impaired host immunity, neurological disability, or other underlying abnormalities, although it may also occur in apparently immunocompetent individuals [4, 17, 36]. In contrast, nodular scabies is generally considered a persistent inflammatory or hypersensitivity reaction to mite antigens and presents with intensely pruritic nodules rather than a high mite burden [8, 17]. Therefore, these two phenotypes should be interpreted separately when evaluating associated abnormalities, complications, and clinical outcomes. Complications, particularly secondary bacterial infections, contribute to significant morbidity and, in some cases, mortality [11].

Scabies, which affects 200–300 million people worldwide, often affects economically disadvantaged and densely populated communities in tropical regions [24]. As scabies mites are transmitted directly or indirectly (through clothing, bedding, etc.), several family members can be affected concurrently, especially if they infect each other [14, 31]. Prolonged and frequent skin contact, along with the number of mites on the skin, increases the risk of scabies transmission [14, 24]. Scabies is also more common in winter than in spring and summer [20].

To prevent transmission and reinfection, all close contacts should be treated simultaneously, even if they are asymptomatic. If left untreated, infected individuals can be a source of reinfection for others. Topical permethrin and oral ivermectin are the most common first-line treatments in the United Kingdom and the United States [26]. In infants under two months, preparations containing 5% sulfur are recommended as an alternative treatment to permethrin [34]. Oral ivermectin can be used in patients who have completed treatment with topical permethrin and benzyl benzoate without success. Fewer treatment failures have been reported with oral ivermectin than topical treatments [37].

This study aimed to describe the demographic characteristics, seasonal distribution, household-related factors, treatment patterns, complications, and 12-month scabies-related re-attendance among children diagnosed with scabies. A specific objective of the analysis was to distinguish crusted scabies from nodular scabies and to evaluate the underlying abnormalities and clinical outcomes associated with each phenotype separately.

## Materials and methods

### Ethics approval

This study was conducted in accordance with the principles of the Declaration of Helsinki. Ethics committee approval was obtained from the Clinical Research Ethics Committee of University of Health Sciences, Haseki Training and Research Hospital (decision No. 64-2023, dated March 29, 2023). Given the retrospective nature of the study and the use of anonymized, routinely collected data, the requirement for informed consent was waived by the ethics committee.

### Study design and setting

This was a single-center, retrospective observational cohort study based on medical record review, with retrospective ascertainment of scabies-related re-attendance within 12 months after the index visit. All pediatric patients (<18 years) with a clinical diagnosis of scabies who presented to our hospital between March 1, 2018 and March 1, 2023 were screened. The study was conducted at a tertiary care academic hospital located in an inner-city setting.

Scabies diagnosis was established clinically by board-certified dermatologists and pediatricians using chart-documented criteria: (i) compatible history (nocturnal pruritus and/or close/household contact with an affected individual); (ii) typical lesion morphology (pruritic papules, excoriations, and/or burrows); and (iii) characteristic distribution on physical examination (e.g., interdigital spaces, wrists, axillae, trunk/waistline, and genital area; in infants, scalp/face and palm/sole involvement was also considered compatible when documented). Dermoscopy and microscopic confirmation (e.g., skin scraping) were not performed routinely in this retrospective dataset; therefore, case ascertainment relied on contemporaneous clinician-documented findings in the medical records. Where possible, diagnoses were mapped to the 2020 International Alliance for the Control of Scabies (IACS) Consensus Criteria during chart review; however, due to the lack of routine confirmatory testing and variability in documentation, cases generally corresponded to the “clinical” or “suspected” IACS categories rather than “confirmed” scabies [12].

### Data sources and variables

Data were extracted retrospectively from the hospital information system. Extracted variables included demographic characteristics, index admission date, presenting complaints, physical examination findings, predominant region of involvement, laboratory results (when available), hospitalization status, treatments received before presentation, treatments recommended by our center, whether a treatment change was required, underlying disease status, number of people in the household, family socioeconomic status, reported use of shared clothing and bedding, and the reported frequency of handwashing and clothes laundering. Information on environmental hygiene and decontamination practices (e.g., laundering of clothing/bedding/towels and related behaviors) was extracted from routine clinical notes in the hospital information system. These variables reflect caregiver self-report as documented in the medical records; adherence and specific laundering conditions (e.g., temperature and duration) were not standardized or objectively verified. Shared use of clothing and shared bed use were coded as yes/no as documented. Laundry/clothes-changing frequency was coded into three categories (<2/week, 2–4/week, >4/week), and “handwashing without soap” was coded as yes/no based on caregiver report documented at the index visit.

Underlying medical issues were coded both as a binary variable (presence or absence of any underlying medical issue) and by diagnostic category. For analyses according to scabies phenotype, underlying abnormalities were grouped as skin disease, immunodeficiency, neurological/developmental disorder, genetic/chromosomal disorder, endocrine/metabolic or nutritional disorder, cardiopulmonary disease, and other chronic systemic conditions. When a patient had more than one underlying condition, each relevant diagnostic category was recorded descriptively.

For age-stratified analyses, patients were categorized into four age groups: <6 months, 6–12 months, 1–5 years, and >5 years. Family socioeconomic status (SES) was abstracted from the social history section of the medical record and categorized as low/medium/high according to our institution’s routine chart-based classification using caregiver-reported monthly household income and parental educational attainment, as documented by the evaluating clinician (and/or social work notes when available). In operational terms, “low” SES corresponded to documentation of low household income and/or primary-school education in caregivers, “medium” SES corresponded to intermediate income/education (secondary/high school), and “high” SES corresponded to higher/steady income and/or university-level education in at least one caregiver.

### Definitions

The index visit was defined as the patient’s first presentation to our hospital for scabies during the study period. Scabies infestation sites were abstracted from the physical examination notes recorded at the index visit. For analysis, we coded the primary (predominant) site of involvement as documented by the examining clinician (i.e., the first-mentioned or most prominent site). Although scabies frequently involves multiple body areas in infants and young children, multisite involvement was not recorded in a standardized manner across all charts; therefore, a single primary site per patient was used for reporting.

Classic scabies was defined as an intensely pruritic eruption with typical lesion morphology and distribution, including papules, excoriations, and/or burrows involving sites such as the interdigital spaces, wrists, axillae, trunk/waistline, and genital area; in infants, scalp/face and palm/sole involvement was also considered compatible when documented. Crusted scabies was defined by clinician-documented thick scaling, hyperkeratotic plaques, crusting, and/or fissuring. Nodular scabies was defined by clinician-documented persistent pruritic nodules attributed to scabies.

Because crusted and nodular scabies represent clinically and immunologically distinct manifestations, descriptive analyses report these phenotypes separately. The combined term “scabies variants” was retained only for selected multivariable regression analyses because nodular scabies was uncommon in the cohort, which limited the stability of separate adjusted estimates.

Secondary bacterial infection was defined as clinician-documented bacterial superinfection, including impetiginization, pustulation, purulent crusting/exudate, or other findings considered by the treating clinician to require antibacterial therapy, based on clinical assessment and available laboratory parameters. Microbiological cultures (blood and/or wound swab) were obtained at the discretion of the treating team and were not required to classify a case as secondary bacterial infection. For patients admitted to the pediatric ward, wound/skin and/or relevant site cultures were routinely obtained at admission; therefore, culture sampling was performed for all hospitalized patients.

Readmission (re-attendance) was defined as any scabies-related re-presentation recorded within 12 months after the index visit. To identify these cases, re-presentations were ascertained retrospectively through chart review until March 1, 2024, providing up to 12 months of follow-up for re-attendance after the index visit. Readmissions following complete recovery after treatment were classified as recurrences. Cases in which patients returned without achieving full recovery, despite a reduction in symptoms and clinical findings, were classified as treatment-resistant or indicative of inadequate treatment. Because routine post-treatment follow-up was not standardized, confirmed clinical recovery could not be assessed for all patients. Therefore, the follow-up outcome was defined as scabies-related re-attendance rather than documented recovery.

### Study population

**Figure 1:**
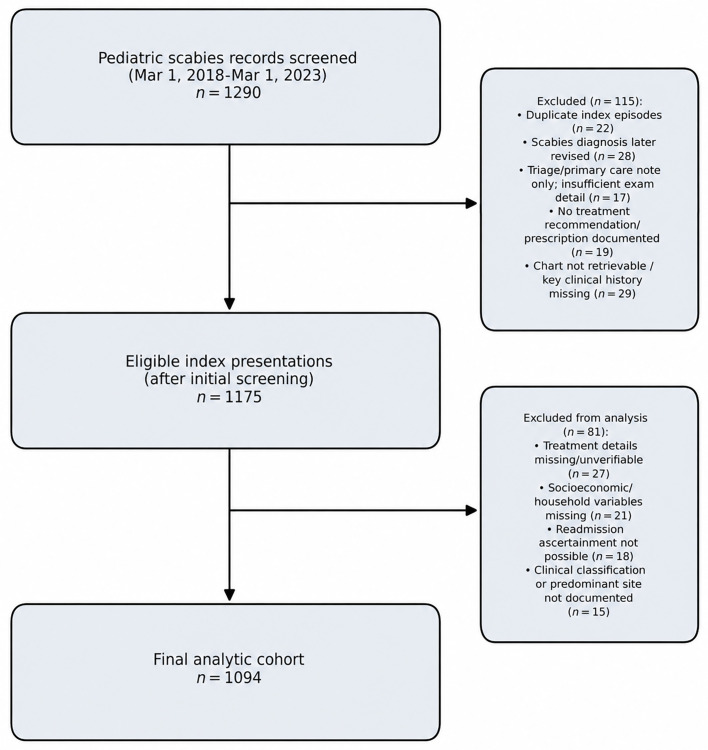
Study flowchart.

Patients with information that was missing or could not be retrieved from the hospital information system were excluded. We initially screened 1,290 pediatric records with a scabies diagnosis during the study period. Of these, 115 records were excluded before eligibility because they represented duplicate index episodes (*n* = 22), the initial scabies diagnosis was later revised after subsequent evaluation (*n* = 28), documentation was limited to triage/primary care notes without sufficient clinical examination detail (*n* = 17), no treatment recommendation or prescription was recorded (*n* = 19), or the chart could not be retrieved / key clinical history was missing (*n* = 29). The remaining 1,175 index presentations constituted the eligible cohort. For the primary analytic dataset, we required availability of key variables for group comparisons and multivariable models. Therefore, an additional 81 records were excluded from analysis due to missing or unverifiable key variables: missing treatment details (*n* = 27), missing socioeconomic/household variables (*n* = 21), inability to ascertain readmission status (*n* = 18), and missing documentation of clinical classification or predominant site (*n* = 15). The study flow is summarized in Figure 1. Patients were categorized into two groups: outpatients (Group 1, *n* = 1,016) and inpatients (Group 2, *n* = 78). Patients were further classified into classic (*n* = 983) and variant scabies (crusted or nodular; *n* = 111) groups.

### Statistical analysis

SPSS 15.0 for Windows (SPSS Inc., Chicago, IL, USA) was used for all statistical analyses. The Shapiro–Wilk test was used to assess the normal distribution of continuous variables. Categorical variables are expressed as number (percentage), and continuous variables are presented as mean (standard deviation) or median (minimum–maximum), as appropriate. Depending on the distribution and cell counts, the chi-square test (or Fisher’s exact test) and the independent-samples *t* test (or Mann–Whitney U test) were used to compare groups.

Multivariate analyses were performed using logistic regression models to identify independent predictors of (i) variant scabies and (ii) inpatient status. Age was entered into the regression models as a continuous variable. Because younger age was associated with both variant scabies and inpatient admission in descriptive analyses, age-related effect estimates are reported per 1-year decrease in age. Variables with significant univariate associations and no evidence of collinearity were entered into the multivariable models. Model goodness-of-fit was assessed using the Hosmer–Lemeshow test. A two-tailed *p*-value of <0.05 was considered statistically significant.

Scabies phenotype was reported descriptively as three categories: classic scabies, crusted scabies, and nodular scabies. Categorical variables were compared across these three phenotypes using Pearson’s chi-square test, Fisher’s exact test, or the Fisher–Freeman–Halton exact test, as appropriate. Because the number of nodular scabies cases was small (*n* = 18), phenotype-specific multivariable models for crusted and nodular scabies were not considered statistically stable. Therefore, for multivariable logistic regression, crusted and nodular scabies were combined as “scabies variants,” while descriptive tables present them separately.

#### Missing data handling

No imputation was performed. Analyses were conducted as complete-case analyses on the final analytic cohort (*n* = 1,094). Of 1,175 eligible index presentations, 81 (6.9%) were excluded from analyses due to missing or unverifiable key variables (e.g., treatment details, SES/household variables, readmission status, or clinical classification), which may introduce selection bias if documentation completeness is associated with exposures or outcomes.

## Results

### Patient demographics, admission time, symptoms, and findings

**Figure 2:**
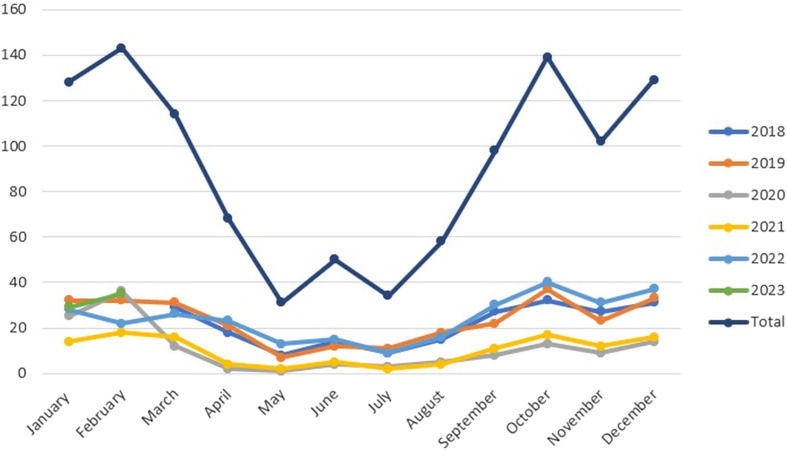
Distribution of admissions of scabies patients by month.

The median age of the 1,094 patients included in the study was 5.2 (1–204) months. Among the patients, 560 (51.2%) were under 6 months of age, 205 (18.7%) were aged 6–12 months, 88 (8%) were between 1–5 years, and 241 (22.1%) were over 5 years old. The demographic and socioeconomic characteristics of the patients are presented in Table 1. Figure 2 displays the distribution of admissions by month. There were 400 cases (36.6%) in winter, 339 (31%) in fall, 213 (19.5%) in spring, and 142 (13%) in summer.

**Table 1:** Demographic and socioeconomic characteristics of patients.

		*n* (%)
Sex	Boy	597 (54.6%)
	Girl	497 (45.4%)
Scabies in the family	(−) No	385 (35.2%)
	(+) Yes	709 (64.8%)
Family size	2–3 individuals	101 (9.2%)
	4–5 individuals	273 (25.0%)
	6–7 individuals	415 (38.0%)
	>7 individuals	305 (27.9%)
Family socioeconomic status	Low	476 (43.5%)
	Medium	512 (46.8%)
	High	106 (9.7%)
Presence of a family member attending school	(−) No	178 (16.3%)
	(+) Yes	916 (83.7%)
Shared use of clothing	(−) No	642 (58.7%)
	(+) Yes	452 (41.3%)
Shared bed use	(−) No	754 (68.9%)
	(+) Yes	340 (31.1%)
Frequency of washing laundry/clothes changing	<2/week	320 (29.3%)
	2–4/week	461 (42.1%)
	>4/week	313 (28.6%)
Handwashing without soap	(−) No	1,070 (97.8%)
	(+) Yes	24 (2.2%)

Underlying medical issues were identified in 12.6% of the patients, with vitamin D deficiency (3.2%) being the most prevalent. Additionally, 4.1% of patients had underlying skin diseases, and 0.9% exhibited immunodeficiency. The most common symptoms were erythema (96%), pruritus (92.2%), and nocturnal itching (55.9%). Among the findings, an erythematous eruption (52.5%) and burrow marks (40.3%) were frequent. The sites of scabies infestations (primary/predominant site) were the extremities (66.3%), trunk (28.1%), and head (5.7%). A concise summary of underlying medical issues according to scabies phenotype is presented in Supplementary Table 1.

### Treatment approaches for patients

Prior to hospital admission, 6.9% of the patients had undergone treatment, with three-quarters of them receiving permethrin therapy. Permethrin was prescribed to 53% of the patients, sulfur-based treatment to 32.4%, and compounded drugs to 19.8%. Additionally, some patients received two different treatments (5.2%). Those under two months of age were exclusively treated with sulfur-based or magistral medications; none received permethrin. The medical records reliably documented the treatment agent prescribed; however, the exact timing and completion of the recommended second application or dose, such as Day 1 plus Day 7–14/15 treatment, were not consistently documented and therefore could not be analyzed as a separate variable.

Among the patients who received treatment, 11.2% (*n* = 123) revisited our hospital. Of these, 118 (95.9%) had used a single type of treatment. Among re-attenders, permethrin was the most frequently documented treatment, accounting for 92.3% of documented treatments. Ninety-two patients (74.8%) were classified as insufficient treatment cases, and 31 (25.2%) as relapse cases. Among insufficient-treatment cases, 92.4% of re-attendances occurred within the first month, whereas among relapse cases, 87.1% of re-attendances occurred between 6 and 12 months.

### Comparison based on scabies types

Of the scabies diagnoses, 89.9% were classic, 8.5% were crusted, and 1.6% were nodular. The median age of patients with classic scabies was 8 months (1–204), and for those with variant scabies, it was 4 months (1–122), a significant difference (*p* < 0.001). Comparisons of demographic and socioeconomic characteristics by hospitalization status and by scabies phenotype are summarized in Table 2. Table 3 presents comparisons of scabies phenotype, predominant site of involvement, treatment patterns, and re-attendance by hospitalization status and by scabies phenotype.

**Table 2:** Comparison of demographic and socioeconomic characteristics by hospitalization status and scabies type.

		Outpatient follow-up (*n* = 1,016) *n* (%)	Inpatient follow-up (*n* = 78) *n* (%)	*p* ^*^	Classic type (*n* = 983) *n* (%)	Crusted type (*n* = 93) *n* (%)	Nodular Type (*n* = 18) *n* (%)	*p* ^**^
Sex	Boy	556 (54.7%)	41 (52.6%)	0.712	529 (53.8%)	56 (60.2%)	12 (66.7%)	0.421^*^
	Girl	460 (45.3%)	37 (47.4%)		454 (46.2%)	37 (39.8%)	6 (33.3%)	
Scabies in the family	(−) No	354 (34.8%)	31 (39.7%)	0.382	347 (35.3%)	30 (32.3%)	8 (44.4%)	0.597^*^
	(+) Yes	662 (65.2%)	47 (60.3%)		636 (64.7%)	63 (67.7%)	10 (55.6%)	
Family size	2–3 individuals	98 (9.6%)	3 (3.8%)	0.087	93 (9.5%)	7 (7.5%)	1 (5.6%)	0.572
	4–5 individuals	254 (25.0%)	20 (25.6%)	0.921	254 (25.8%)	16 (17.3%)	4 (22.2%)	
	6–7 individuals	391 (38.6%)	25 (32.0%)	0.166	374 (38.0%)	35 (37.6%)	7 (38.9%)	
	>7 individuals	273 (26.9%)	30 (38.4%)	**0.007**	262 (26.7%)	35 (37.6%)	6 (33.3%)	
Family socioeconomic status	Low	454 (44.7%)	44 (56.4%)	**0.045**	430 (43.8%)	60 (64.5%)	8 (44.5%)	**<0.001**
	Medium	466 (45.9%)	29 (37.2%)	0.137	479 (48.7%)	10 (10.8%)	6 (33.3%)	
	High	96 (9.4%)	5 (6.4%)	0.376	74 (7.5%)	23 (24.7%)	4 (22.2%)	
Presence of a family member attending school	(−) No	175 (17.2%)	3 (3.8%)	**0.002**	159 (16.2%)	15 (16.2%)	4 (22.2%)	0.673
	(+) Yes	841 (82.8%)	75 (96.2%)		824 (83.8%)	78 (83.8%)	14 (77.8%)	
Shared use of clothing	(−) No	611 (60.1%)	31 (39.7%)	**<0.001**	580 (59.0%)	52 (55.9%)	10 (55.6%)	0.747^*^
	(+) Yes	405 (39.9%)	47 (60.3%)		403 (41.0%)	41 (44.1%)	8 (44.4%)	
Shared bed use	(−) No	719 (70.8%)	35 (44.9%)	**<0.001**	676 (68.8%)	67 (72.0%)	11 (61.1%)	0.646^*^
	(+) Yes	297 (29.2%)	43 (55.2%)		307 (31.2%)	26 (28.0%)	7 (38.9%)	
Frequency of washing laundry/clothes changing	<2/week	287 (28.2%)	33 (42.3%)	**0.008**	291 (29.6%)	23 (24.7%)	6 (33.3%)	0.165
	2–4/week	435 (42.8%)	26 (33.3%)	0.101	420 (42.7%)	33 (35.5%)	8 (44.4%)	
	>4/week	294 (28.9%)	19 (24.4%)	0.397	272 (27.7%)	37 (39.8%)	4 (22.2%)	
Handwashing without soap	(−) No	996 (98.0%)	74 (94.9%)	0.073	964 (98.1%)	89 (95.7%)	17 (94.4%)	0.125^*^
	(+) Yes	20 (2.0%)	4 (5.1%)		19 (1.9%)	4 (4.3%)	1 (5.6%)	

^*^Chi-square (Fisher) Test, ^**^Fisher–Freeman–Halton exact test.

**Table 3:** Comparison of scabies type, location, and treatment by hospitalization status and scabies classification.

		Outpatient follow-up (*n* = 1,016) *n* (%)	Inpatient follow-up (*n* = 78) *n* (%)	*p* ^*^	Classic type (*n* = 983) *n* (%)	Crusted type (*n* = 93) *n* (%)	Nodular type (*n* = 18) *n* (%)	*p* ^**^
Scabies type	*Classic type*	953 (93.8%)	30 (38.4%)	**<0.001**	983 (100.0%)	0 (0%)	0 (0%)	
	*Crusted type*	55 (5.4%)	38 (48.7%)	**<0.001**	0 (0%)	93 (100%)	0 (0%)	**<0.001**
	*Nodular type*	8 (0.8%)	10 (12.8%)	**<0.001**	0 (0%)	0 (0%)	18 (100%)	
Predominant site of involvement	*Head*	58 (5.7%)	4 (5.1%)	0.057	56 (5.7%)	5 (5.4 %)	1 (5.6%)	
	*Trunk*	294 (28.9%)	13 (16.7%)		266 (27.0%)	35 (37.6%)	6 (33.3%)	0.241
	*Extremities*	664 (65.4%)	61 (78.2%)		661 (67.2%)	53 (57.0%)	11 (61.1%)	
Pre-admission treatment		64 (6.3%)	11 (14.1%)	**0.009**	21 (2.1%)	50 (53.7 %)	4 (22.2%)	**<0.001**
	*Permethrin*	46 (71.8%)	7 (63.6%)	0.440	18 (85.7%)	32 (64.0%)	3 (75.0%)	
	*Sulfur-based*	13 (20.3%)	2 (18.2%)	0.870	2 (9.5%)	12 (24.0%)	1 (25.0%)	
	*Compounded (magistral)*	5 (7.8%)	2 (18.2%)	0.272	1 (4.8%)	6 (12.0%)	0 (0%)	
Treatment prescribed by the pediatrician^†^	*Permethrin*	537 (52.9%)	43 (55.1%)	0.689	559 (56.9%)	15 (16.1%)	6 (33.3%)	**<0.001**
	*Sulfur-Based*	334 (32.9%)	20 (25.6%)	0.188	318 (32.3%)	28 (30.1%)	8 (44.4%)	
	*Compounded (magistral)*	187 (18.4%)	30 (38.5%)	**<0.001**	175 (17.8%)	38 (40.9%)	4 (22.2%)	
	*Oral ivermectin*	22 (2.2%)	18 (23.1%)	**<0.001**	0 (0%)	39 (41.9%)	1 (5.6%)	
Re-attendance		120 (11.8%)	3 (3.8%)	**0.032**	77 (7.8%)	44 (47.3%)	2 (11.1%)	**<0.001**

^*^Chi-square (Fisher) Test, ^**^Fisher–Freeman–Halton exact test.

^†^Some patients received more than one treatment modality; therefore, categories are not mutually exclusive and percentages may exceed 100%.

^***^Treatment categories refer to documented treatment agents prescribed or recorded in the medical chart. Completion of a second application or dose at Day 7–14/15 and simultaneous treatment of household contacts were not consistently documented; therefore, adherence to the recommended double-treatment schedule could not be tabulated.

Underlying medical issues and specific diagnoses were evaluated separately according to scabies phenotype and are presented in Supplementary Table 1. Among patients with crusted scabies, the most frequent recorded abnormalities were vitamin D deficiency (*n* = 10, 10.8%), type 1 diabetes mellitus (*n* = 7, 7.5%), asthma (*n* = 6, 6.5%), epilepsy (*n* = 4, 4.3%), cerebral palsy (*n* = 4, 4.3%), and dermatitis (*n* = 4, 4.3%). Down syndrome was documented in two patients, both of whom had crusted scabies. Among patients with nodular scabies, the most frequent recorded abnormalities were seasonal allergic rhinitis (*n* = 5, 27.8%) and allergic urticaria (*n* = 4, 22.2%). These findings indicate that the underlying abnormality profile differed between crusted and nodular scabies.

Oral ivermectin was not administered to any patient weighing less than 15 kg. Oral ivermectin was prescribed predominantly in crusted scabies (39/93, 41.9%) and rarely in nodular scabies (1/18, 5.6%). Among the two hospitalized patients with nodular scabies, one (50%) received intralesional corticosteroid treatment.

### Comparisons based on hospitalization status

The median age differed significantly between outpatients and inpatients, with values of 6.6 (1–204) months and 3.6 (1–56) months, respectively (*p* < 0.001). The average hospital stay length for admitted patients, excluding those followed up on an outpatient basis, was 6.2 ± 2.6 days.

Clinically suspected secondary bacterial infection was documented in 77 of the 78 hospitalized patients, based on clinician-recorded symptoms and physical examination findings. Among hospitalized patients, 48 of 78 children (61.5%) had variant scabies, which was significantly more frequent than among outpatients (*p* < 0.001). Underlying diseases were present in 42 patients (53.8%), with 37 having skin conditions and 5 with immunodeficiencies. Blood and swab cultures were obtained from all hospitalized patients (*n* = 78), and culture results were positive in 27 patients (34.6%), with methicillin-resistant *Staphylococcus aureus* detected in 10 cases, methicillin-sensitive *S. aureus* in 7, *Streptococcus* in 4, other *Staphylococcus* species in 3 cases, and other organisms in 3 cases. The three most commonly administered treatments were ampicillin-sulbactam (81.5%), clindamycin (63.0%), and ceftriaxone (14.8%). None of the patients required a change in antibiotic therapy. Among the inpatients, the only one not hospitalized for a secondary bacterial infection was an individual who had tested positive for severe acute respiratory syndrome coronavirus 2.

### Independent predictors of variant scabies and inpatient admission

We used multivariable logistic regression models for two analyses. The first predicted variant scabies by incorporating demographic factors (e.g., age, gender, and family size), socioeconomic characteristics, seasonal variations, and underlying diseases. Among these variables, underlying disease and younger age were identified as independent predictors of variant scabies (odds ratio [OR]: 6.87, 95% confidence interval [CI]: 3.66–10.24, *p* < 0.001; and OR per 1-year decrease in age: 1.56, 95% CI: 1.05–2.41, *p* = 0.022, respectively). Other candidate covariates entered into the model did not retain statistical significance after adjustment.

The second model was used to predict inpatient admission and included similar variables: demographic characteristics, socioeconomic status, seasonal variations, scabies type, and underlying diseases. Underlying disease, variant scabies, and younger age emerged as independent predictors of hospitalization (OR: 1.32, 95% CI: 1.07–2.42, *p* = 0.031; OR: 1.91, 95% CI: 1.05–2.94, *p* = 0.017; and OR per 1-year decrease in age: 2.07, 95% CI: 1.44–3.15, *p* = 0.008, respectively).

## Discussion

Our findings support the importance of distinguishing crusted scabies from nodular scabies in pediatric cohorts. Crusted scabies was the predominant non-classic phenotype and was associated with a distinct profile of underlying abnormalities and greater use of systemic or combined treatment approaches. In contrast, nodular scabies was uncommon and showed a different pattern of associated conditions, with allergic disorders being more prominent in the descriptive table. Therefore, crusted and nodular scabies should not be interpreted as equivalent manifestations. The combined “scabies variants” category was retained only for selected regression analyses because the nodular subgroup was small, but the descriptive results are presented separately to avoid biologically misleading interpretation.

Similar to other studies, male prevalence was higher in our study [13, 22], and the age distribution indicated that scabies occurred most frequently in infants younger than 6 months and was also common in children older than 5 years [10, 19]. Because our cohort covered the full pediatric age range, our findings provide complementary information to previous studies focused mainly on preschool- or school-aged children. This marked predominance of infants <6 months likely reflects the case mix and referral patterns of our tertiary inner-city center, where caregivers have a lower threshold to seek medical attention for very young infants and clinicians more consistently document scabies when severe pruritus and/or household exposure is reported. In addition, infants may present with more widespread or atypical involvement (including scalp/face or palms/soles), which can prompt earlier presentation and more detailed documentation. These system-level factors may partially explain the higher proportion of infants in our cohort compared with community-based studies focused on preschool or school-aged populations.

Previous studies have indicated that scabies cases rise during the fall and winter months due to changes in temperature and humidity, which was also observed in our study [20, 21, 29]. Additionally, increased time spent indoors, such as at school and home, and closer contact with others may have contributed to higher transmission rates during these months. In our study, the number of cases decreased in 2020 and 2021, likely due to the new coronavirus outbreak, before rising again in subsequent years. Despite the increase in domestic transmission during the quarantine period, patients did not seek medical attention until the itching became unbearable, which may help to explain the discrepancy in increased transmission and a decrease in the number of cases [27, 39].

In schoolchildren, scabies spreads rapidly due to close contact and overcrowding in schools. Studies have identified various risk factors for scabies transmission, including intrafamilial transmission, crowded households, low socioeconomic status, the presence of pets, sharing clothes and bedding, frequency of handwashing, and soap usage habits [1, 16, 18, 22]. In unadjusted comparisons, variant scabies was more frequently observed in younger children and in families with larger household size and lower socioeconomic status. Likewise, inpatient admission was more common when a household member attended school and when shared use of clothing or bed was reported. However, in the multivariable models, only underlying disease and age remained independent predictors of variant scabies, whereas underlying disease, scabies type, and age remained independent predictors of hospitalization; therefore, household/lifestyle variables should be interpreted as associative findings rather than independent effects. While soap usage during handwashing did not differ significantly between study groups, the handwashing variable was based on caregiver report and was not independently associated with the main outcomes; therefore, no firm inference can be made regarding its protective effect [16].

Permethrin is the first-line topical drug for scabies in the United Kingdom and the United States [6]. The United States Food and Drug Administration has approved 5% permethrin cream for use during pregnancy and lactation, and in children aged 2 months and older [24]. In our study, while sulfur and magistral treatments were more frequently prescribed for variant scabies cases prior to hospital admission, permethrin remained the most commonly prescribed treatment in the classic and variant scabies groups. Pediatricians prescribed permethrin less often, possibly reflecting prior permethrin exposure or suspected inadequate response in some patients, prompting consideration of alternative topical options. Pediatricians may also have identified variant scabies more often and chosen non-permethrin options accordingly. Notably, oral ivermectin was exclusively prescribed for variant scabies cases, reflecting its recommendation for combined use with topical treatments in these cases [24]. Despite being classified by the World Health Organization as a safe and effective therapeutic agent [34], ivermectin is not advised for pregnant women or young children (<5 years or < 15 kg) due to limited safety data [25, 30]. National guidelines recommend initiating treatment with local therapies before considering oral ivermectin. In our study, oral ivermectin was more commonly used in hospitalized patients. For infants younger than 2 months, 5% sulfur preparations were recommended as an alternative to permethrin [26], and no infant under this age received permethrin or oral ivermectin in our study. When treatment was evaluated according to phenotype, oral ivermectin use was concentrated in crusted scabies rather than nodular scabies, which is consistent with the need for more intensive or combined therapy in high-burden disease. Nodular scabies, by contrast, generally requires management of persistent inflammatory nodules and pruritus rather than escalation based on mite burden alone.

In guidelines, topical permethrin and, in selected cases, oral ivermectin are recommend as part of scabies management [3, 28, 35]. A key component of effective scabies treatment is repeat treatment after the initial application or dose, commonly performed as a second application or dose approximately 7–14 days later, including Day 1 plus Day 15 regimens in some protocols. This approach is intended to target mites that may emerge after the initial treatment and to reduce persistent infestation, treatment failure, and reinfestation. In retrospective records, however, we could determine the prescribed treatment agent more reliably than whether the second application or dose was completed as instructed. In our cohort, permethrin accounted for 92.3% of documented treatments among children who re-presented within one year. This finding should be interpreted in the context of routine prescribing patterns, as permethrin is commonly used as first-line therapy, whereas alternative agents are typically reserved for selected patients (e.g., prior treatment exposure, suspected inadequate response, or crusted/nodular variants). Therefore, the treatment distribution observed among re-attenders does not, by itself, indicate that permethrin is associated with a higher risk of recurrence. True recovery rates could not be calculated in this retrospective cohort because routine post-treatment control visits were not standardized and clinical resolution was not consistently documented for patients who did not re-attend. Consequently, our follow-up data reflect scabies-related re-attendance, treatment-resistant or insufficient-treatment presentations, and relapse among those who returned to our hospital, rather than confirmed recovery in the entire cohort. Readmissions in scabies are multifactorial and may reflect reinfestation due to incomplete treatment of close contacts, suboptimal application of topical therapy, and/or persistent infestation sources in the household environment [32]. Importantly, Aussy *et al.* [3] emphasized inadequate decontamination of furnishings and the home environment as a key contributor to treatment failure and recurrence. In our retrospective cohort, these determinants could not be objectively assessed; available information was limited to caregiver-reported behaviors documented in the charts (e.g., shared clothing/bedding and reported washing frequency), and thus their contribution to readmissions could not be quantified. The lower readmission rate among hospitalized patients may relate to supervised treatment delivery and more structured caregiver counseling during hospitalization. The higher readmission rate observed in the variant scabies group may also be influenced by limited use of combined treatment approaches; in our country, stepwise or combination regimens are generally reserved for patients who fail initial local therapy rather than being used upfront [40].

Crusted and nodular scabies together accounted for 10.1% of the cohort; however, these phenotypes require separate interpretation. Crusted scabies is generally associated with impaired host defense, neurological disability, or other underlying abnormalities that may permit a high mite burden [4, 17, 36]. This is consistent with our observation that chronic systemic, neurological/developmental, and dermatological conditions were more prominent among children with crusted scabies. By contrast, nodular scabies is better understood as a persistent inflammatory or hypersensitivity response to mite antigens rather than a marker of heavy mite burden or impaired immunity [8, 17]. In our cohort, nodular scabies was rare, and allergic conditions were relatively more frequent in this subgroup; therefore, these findings should be interpreted descriptively rather than as evidence of an immunodeficiency-associated phenotype [4, 8, 17, 36]. Clinically suspected secondary bacterial infection was documented in nearly all hospitalized patients, and variant scabies was overrepresented among hospitalized children. However, because secondary bacterial infection was mainly ascertained in the inpatient setting, this association should be interpreted in the context of local admission practices and case mix. This pattern likely reflects local admission practice and case mix in our center, where hospitalization is preferentially used for children requiring closer monitoring and management of complications. This context should be considered when interpreting hospitalization-associated findings. Although low culture yield has been reported in deeper soft-tissue infections such as cellulitis and erysipelas [15], this evidence should not be extrapolated to scabies-associated superficial bacterial involvement. The modest culture positivity in our cohort should therefore be interpreted cautiously. Because secondary bacterial involvement in scabies is often superficial and may be difficult to distinguish clinically from excoriated or inflamed scabies lesions, negative cultures in a substantial proportion of clinically suspected cases raise the possibility that some cases did not represent true secondary bacterial infection. Therefore, culture results were considered supportive rather than definitive, and secondary bacterial infection in this retrospective cohort should be interpreted as a clinically suspected diagnosis unless microbiologically confirmed. In comparable studies, *S. aureus* is the most frequently observed pathogen in secondary bacterial infections [9, 19]. Regional variations in methicillin-resistant *Staphylococcus* rates have also been noted. In countries including the United States and Turkey, where methicillin-resistant strains are more prevalent, empirical treatment must account for this resistance [5, 33, 38]. This may explain the high rates of clindamycin use observed in our study.

### Study limitations

The primary limitation of our investigation was the retrospective study design, which precluded access to several potentially important variables. In addition, the hospitalized subgroup was relatively small (*n* = 78), which limits statistical power and the precision of estimates in subgroup comparisons and multivariable modeling (i.e., wider confidence intervals and potential model instability). Although crusted and nodular scabies were presented separately in the descriptive analyses, the nodular scabies subgroup was small (*n* = 18), which limited statistical power and precluded stable phenotype-specific multivariable modeling. In addition, clinical recovery after treatment could not be systematically assessed because routine follow-up visits and standardized documentation of symptom and lesion resolution were not available for all patients. Re-attendance was ascertained only within our hospital information system; therefore, scabies-related visits to other healthcare facilities could not be captured, which may have led to underestimation of the true re-attendance rate. This limitation is inherent to single-center, record-based follow-up and may affect the generalizability of recurrence estimates. Key information, such as whether treatment instructions were adequately explained, whether all household members received treatment simultaneously, whether the second application or dose was completed at Day 7–14/15, and adherence to recommended environmental measures, could not be reliably determined from the records. In addition, certain patient-level characteristics (e.g., nutritional status, including malnutrition or obesity) and household factors (e.g., nail clipping frequency, pet ownership, and feeding practices at home) were not consistently documented. Measures needed to grade disease severity (e.g., extent of skin involvement/lesion burden) were also not available in a standardized format. A further limitation is that scabies was diagnosed clinically without routine dermoscopic or microscopic confirmation; therefore, some diagnostic misclassification is possible, particularly in atypical presentations or in patients with concurrent dermatoses. We attempted to contextualize case ascertainment using the 2020 IACS Consensus Criteria as a framework for chart review; however, given the retrospective nature of the study and variability in documentation, diagnoses could not be uniformly re-adjudicated from records alone. Accordingly, the contemporaneous diagnosis recorded by the evaluating pediatrician/dermatologist at the time of presentation was accepted as the reference standard [12]. Infestation sites were derived from routine clinical documentation; multisite involvement could not be analyzed because it was not recorded in a standardized manner across all charts. Environmental decontamination and laundering practices were based on caregiver-reported information recorded in the medical records and could not be objectively confirmed; therefore, misclassification is possible. Secondary bacterial infection ascertainment was primarily clinical and not based on a standardized prospective protocol. Although cultures were obtained from hospitalized patients, a substantial proportion showed no growth; therefore, some clinically suspected cases may have represented excoriated, inflamed, or impetiginized scabies lesions rather than microbiologically confirmed secondary bacterial infection. Finally, data on the financial burden and productivity loss associated with the disease could not be estimated within the scope of the available records.

## Conclusions

Crusted scabies and nodular scabies should be distinguished in pediatric scabies cohorts because they appear to reflect different clinical and immunological patterns. Children with crusted scabies require careful assessment for underlying abnormalities, secondary bacterial infection, and the need for more intensive treatment, whereas nodular scabies should be interpreted primarily as a persistent inflammatory or hypersensitivity phenotype. Younger age and underlying disease were independently associated with greater clinical burden, while large family size and low socioeconomic status should be interpreted as contextual associations. Topical permethrin (≥2 months) remains first-line therapy, whereas oral ivermectin should be reserved for selected cases in accordance with weight-/age-related safety recommendations and local guidelines. Treatment plans should be tailored to account for the country’s bacterial resistance patterns to effectively manage secondary infections.

## Data Availability

The authors confirm that data supporting the findings of this study are available within the article. Other data in this study are available from the corresponding author upon reasonable request.

## Supplementary material

*Supplementary Table 1*: Underlying medical issues and specific diagnoses according to scabies phenotype. The supplementary material of this article is available at https://www.parasite-journal.org/10.1051/parasite/2026041/olm.
