# Supplementary material for: Evaluation of 1,094 children diagnosed with scabies in Turkey: a 5-year retrospective observational study
Source: Parasite. 2026 Jul 31;33:42. doi: 10.1051/parasite/2026041 (PMC13432915; doi:10.1051/parasite/2026041)
Supplement: Supplementary file 1 — Supplementary Table 1: Underlying medical issues and specific diagnoses according to scabies phenotype. [file parasite-33-42-s1.pdf]

Supplementary Table 1: Underlying medical issues and specific diagnoses according to scabies phenotype

		n (%)			
**Underlying medical issue**	**(-) Absence**	**956 (87.4%)**			
	**(+) Presence**	**138 (12.6%)**			
	**Total**	**Classic type (n=983) n, (%)**	**Crusted type (n=93) n, (%)**	**Nodular Type (n=18) n, (%)**	***p****
**Vitamin D deficiency**	35 (3.2%)	24 (2.4%)	10 (10.8%)	1 (5.6%)	**<0.001**
**Dermatitis**	32 (2.9%)	27 (2.8%)	4 (4.3%)	1 (5.6%)	0.312
**Immunodeficiency**	8 (0.8%)	0	8 (8.6%)	0	**<0.001**
**Allergic urticaria**	7 (0.7%)	3 (0.3%)	0	4 (22.2%)	**<0.001**
**Asthma**	7 (0.7%)	1 (0.1%)	6 (6.5%)	0	**<0.001**
**Type 1 diabetes mellitus**	7 (0.7%)	0	7 (7.5%)	0	**<0.001**
**Seasonal allergic rhinitis**	6 (0.6%)	1 (0.1%)	0	5 (27.8%)	**<0.001**
**Epilepsy**	4 (0.4%)	0	4 (4.3%)	0	**<0.001**
**Cerebral palsy**	4 (0.4%)	0	4 (4.3%)	0	**<0.001**
**Precocious puberty**	4 (0.4%)	1 (0.1%)	3 (3.2%)	0	**0.005**
**Acne vulgaris**	2 (0.2%)	2 (0.2%)	0	0	1.000
**Atopic dermatitis**	2 (0.2%)	1 (0.1%)	0	1 (5.6%)	0.040
**Atrial septal defect**	2 (0.2%)	1 (0.1%)	1 (1.1%)	0	0.193
**Down syndrome**	2 (0.2%)	0	2 (2.2%)	0	0.010
**Hydrocephalus**	2 (0.2%)	0	2 (2.2%)	0	0.010
**Vertigo**	2 (0.2%)	2 (0.2%)	0	0	1.000
**Hypothyroidism**	2 (0.2%)	0	2 (2.2%)	0	0.010
**Acute rheumatic fever**	1 (0.1%)	0	1 (1.1%)	0	0.101
**Alopecia areata**	1 (0.1%)	0	0	1 (5.6%)	0.016
**Acute rheumatic fever carditis**	1 (0.1%)	0	1 (1.1%)	0	0.101
**Attention deficit and hyperactivity disorder**	1 (0.1%)	1 (0.1%)	0	0	1.000
**Eczema**	1 (0.1%)	0	0	1 (5.6%)	0.016
**Nocturnal enuresis**	1 (0.1%)	1 (0.1%)	0	0	1.000
**Familial Mediterranean fever**	1 (0.1%)	0	1 (1.1%)	0	0.101
**Developmental delay**	1 (0.1%)	0	1 (1.1%)	0	0.101
**Mild mental retardation**	1 (0.1%)	0	1 (1.1%)	0	0.101
**Hyperlepsia**	1 (0.1%)	0	1 (1.1%)	0	0.101
**Hypoplastic left heart**	1 (0.1%)	0	1 (1.1%)	0	0.101
**Undescended testis**	1 (0.1%)	1 (0.1%)	0	0	1.000
**Insulin resistance**	1 (0.1%)	0	1 (1.1%)	0	0.101
**Operative pulmonary binding**	1 (0.1%)	0	1 (1.1%)	0	0.101
**Chronic urticaria**	1 (0.1%)	0	1 (1.1%)	0	0.101
**Lichen simplex chronicus**	1 (0.1%)	0	1 (1.1%)	0	0.101
**Mastocytosis**	1 (0.1%)	0	1 (1.1%)	0	0.101
**Autism**	1 (0.1%)	0	1 (1.1%)	0	0.101
**Autoimmune thyroiditis**	1 (0.1%)	0	1 (1.1%)	0	0.101
**Renal ectasia**	1 (0.1%)	0	1 (1.1%)	0	0.101
**Spina bifida**	1 (0.1%)	0	1 (1.1%)	0	0.101
**Turner syndrome**	1 (0.1%)	0	1 (1.1%)	0	0.101
**Umbilical hernia**	1 (0.1%)	1 (0.1%)	0	0	1.000
**Vesicoureteral reflux**	1 (0.1%)	1 (0.1%)	0	0	1.000

Specific underlying diagnoses were not mutually exclusive; therefore, the sum of individual diagnoses exceeds the number of patients with any underlying medical issue because some patients had more than one condition.

*Fisher–Freeman–Halton exact test
